# NKT Cells in Sepsis

**DOI:** 10.1155/2010/414650

**Published:** 2010-10-04

**Authors:** Briana Leung, Hobart W. Harris

**Affiliations:** Department of Surgery, University of California, San Francisco, San Francisco, CA 94143, USA

## Abstract

Sepsis is currently a leading cause of death in hospital intensive care units. Previous studies suggest that the pathophysiology of sepsis involves the hyperactivation of complex proinflammatory cascades that include the activation of various immune cells and the exuberant secretion of proinflammatory cytokines by these cells. Natural killer T-cells (NKTs) are a sublineage of T cells that share characteristics of conventional T cells and NK cells and bridge innate and adaptive immunity. More recently, NKT cells have been implicated in microbial immunity, including the onset of sepsis. Moreover, apolipoprotein E (apoE), a component of triglyceride-rich lipoproteins, has been shown to be protective in endotoxemia and gram-negative infections in addition to its well-known role in lipid metabolism. Here, we will review the role of NKT cells in sepsis and septic shock, the immunoregulatory role of apoE in the host immune response to infection, and propose a mechanism for this immunoregulation.

## 1. Introduction

Natural killer T (NKT) cells are a heterogeneous and conserved lineage of T cells that have been implicated in tumor immunity, autoimmune diseases such as diabetes and multiple sclerosis, as well as the overall regulation of the immune system [1]. They were originally defined in mice as a double-negative cell population (CD4-CD8-) that coexpresses a T cell receptor (TCR) and NK1.1, a natural killer (NK) cell surface marker [2]. However, subsequent studies revealed that this earlier definition was not entirely accurate and was overly simplified. This is partly due to the lack of NK1.1 in many commonly used mouse strains except the C57BL/6 strain, as well as the discovery of a distinct subset of NK1.1- cells that also exhibited key defining characteristics of NKT cells [2, 3].

## 2. Classification and Characteristics of NKT Cells

Currently, it is proposed that NKT cells can be broadly categorized into two groups: type I or invariant NKT (iNKT) cells and type II NKT cells [3]. Both groups are activationally restricted by the MHC class I-like molecule called CD1d [3]. CD1d is a member of a family of CD1 glycoprotein molecules expressed on various antigen-presenting cells (APCs) associated with *β*_2_-microglobulin [4]. Unlike MHC class I and II molecules that present peptide antigens, CD1d can bind and present glycolipid and lipid antigens for recognition by NKT cells [4]. Because many pathogenic microbes possess lipids and glycolipids as structural elements (e.g., lipopolysaccharide), the discovery of a potential system for T cell-mediated recognition of these macromolecules held large implications for host microbial immunity. 

A major difference between type I and II NKT cells lays in the components of their T cell receptors (TCRs) and their known ligands. iNKT cells express semi-invariant *α*/*β* T cell receptors (TCRs) consisting of an invariant *V* *α*14/*J* *α*18 chain in mice (*V* *α*24/*J* *α*18 in humans) and a restricted *β* chain [3]. Despite having well-characterized TCRs, the endogenous ligands of iNKT cells are still unknown. One potential lipid proposed to be an *in vivo* ligand of iNKT cells is isoglobotrihexosylceramide (iGb3). iGb3, in conjunction with CD1d, has been implicated in the positive selection of iNKT cells in the thymus as well as in the peripheral activation of iNKT cells by dendritic cells (DCs) [5]. However, there is lack of evidence that iGb3 is expressed in mouse or human thymus, and mice deficient in iGb3 synthase still experience normal iNKT cell development and function [5, 6]. This suggests that iGb3 is unlikely to be the endogenous lipid ligand responsible for thymic iNKT cell selection and activation. 

In the absence of physiological ligands, alpha-galactosyl-ceramide (*α*-GalCer), a synthetic glycosphingolipid (GSL) derived from marine sponges, is the most potent known agonist of iNKT cells and an indispensable tool in studying the impact of NKT cell activation on microbial immunity [7, 8]. In contrast to iNKT cells, type II NKT cells are nonresponsive to *α*-GalCer and possess a more diverse TCR repertoire [3]. Sulfatide, a self-glycolipid derived from myelin, has been shown to bind to murine CD1d and be recognized by a subset of type II NKT cells [9, 10]. It has been suggested that type II NKT cells activated by sulfatide treatment can mediate anergy in iNKT cells, and thus, have potential therapeutic applications for inflammatory diseases in which iNKT cells have been strongly implicated, including antitumor immunity, autoimmune disease, and asthma [11].

Despite increasing research on type II NKT cells, relatively little is known about them. iNKT cells remain the prototypical NKT cell type more extensively studied. The immunoregulatory function most characteristic of NKT cells is their ability to promptly secrete large amounts of Th1 and Th2 cytokines including IFN-*γ* and IL-4, respectively, upon stimulation [12, 13]. Downstream, this culminates in the activation of cell types of the innate immune system such as macrophages, NK cells, and dendritic cells as well as effector T cells of the adaptive immune system. In addition to cytokine production, NKT cells also possess cytotoxic effector activity by way of lysis of target cells that is dependent on perforin and FasL [14, 15]. Although shown to mediate immunity against a wide range of pathogenic microbes, including bacteria, fungi, parasites, and viruses, the mechanism(s) by which NKT cells are activated during infection is still unclear [16]. Nevertheless, it is this ability to mount rapid responses to a variety of pathogens and subsequently activate other cell types that indicates a critical role for NKT cells in bridging innate and acquired immunity.

## 3. iNKT Cells Contribute to the Pathogenesis of Sepsis

Sepsis is a life-threatening condition that continues to be a chief cause of death in intensive care units [17]. Despite over two decades of research, the pathogenesis of sepsis is still incompletely understood, and there are no effective therapies beyond supportive care and antibiotics. In sepsis, systemic exposure to pathogenic microbial lipids initiates a complex and dysregulated immune response. Previous studies have provided evidence that this immune response consists of an initial hyperreactive phase and a latent phase. The initial hyperreactive phase is characterized by the large release of pro-inflammatory cytokines (i.e., tumor necrosis factor (TNF), IL-1, and IFN-*γ*) from activated monocytes, macrophages, and other immune cells. The latent anti-inflammatory or immunosuppressed phase increases in strength in the late stages of sepsis. This latter phase is characterized by hypotensive shock, inability to clear the infection, increased susceptibility to nosocomial infections, and possible multiple organ failure and death [18]. Because iNKT cells are potent producers of IFN-*γ* and other pro-inflammatory mediators, they were thought to be significant promoters of the dysregulated septic response. To better understand how iNKT cells contribute to the pathophysiology of sepsis, researchers have investigated the role of these cells in various experimental models that mimic the clinical signs and symptoms of sepsis (Table 1).

## 4. Endotoxic Shock and Gram-Negative Bacterial Infections

Lipopolysaccharide (LPS), the endotoxin of gram-negative bacteria, has long been considered a primary structural component of bacteria that is responsible for initiating the septic inflammatory response [27]. LPS binds to CD14 expressed on the surface of macrophages and other myeloid lineage cells, triggering an intracellular signal that is dependent on toll-like receptor (TLR), and ultimately leads these cells to secrete many pro-inflammatory mediators [28–31]. Administration of nanogram quantities of LPS to patients was found to reproduce clinicopathological signs of sepsis including the activation of complement and coagulation cascades as well as the increased release of pro-inflammatory cytokines [32]. Intravenous injection of larger doses of endotoxin can lead to hypotensive shock and multiple organ dysfunction in a matter of hours [33]. In light of this relationship between LPS and the development of a host septic response, LPS has been commonly used to generate experimental models of sepsis.

One such model is the generalized Shwartzman reaction, a lethal shock syndrome in mice that is induced by two consecutive injections of LPS: a priming followed by a challenging dose. IFN-*γ* is considered a key cytokine in the pathogenesis of the generalized Shwartzman reaction because IFN-*γ* can elicit the large production of TNF-*α*, IL-1, and other inflammatory mediators [34–36]. Recent studies have found that because it induces IFN-*γ*, IL-12 is crucial for the priming phase. In fact, IL-12 and IFN-*γ* reportedly can replace the LPS priming dose in sensitizing mice and producing significant mortality after further challenge with LPS [35, 36]. APC-derived IL-12 strongly activates NK1+ T cells to promptly secrete vast amounts of IFN-*γ* [37]. In addition, LPS activates and increases the cytotoxicity of NK1.1+ T cells in the liver through IL-12 produced by Kupffer cells [38]. These findings thus influenced additional studies on the contribution of NKT cells to the development of lethal endotoxic shock. In mice depleted of NK1+*α* *β* T cells and NK cells by anti-NK1.1 Ab, and in mice deficient in B2-microglobulin (*β*2m^−/−^) and lacking most of their NK1+*α* *β* T cells, IFN-*γ* production was reduced after IL-12 priming, and consequently, the mice were protected against mortality upon subsequent LPS challenge [19]. Similarly, a study that examined the systemic Shwartzman reaction in iNKT cell-deficient (*J* *α*281-deficient) mice found that when primed with either LPS or IL-12, they had a survival advantage and concurrently lower serum levels of IFN-*γ* and TNF-*α* than did wild-type C57BL/6 mice [20]. Activation of iNKT cells alone by injection of the glycolipid *α*-GalCer was recently shown to effectively replace the LPS priming dose in the generalized Shwartzman reaction [39]. Taken together, these findings indicate a primary role for NKT cells in initiating an excessive pro-inflammatory response and promoting lethality in endotoxic shock.

Given that the development of endotoxic shock appears to be driven by disproportionate Th1 cytokine secretion, studies have investigated the therapeutic potential of *α*-GalCer in influencing the Th1/Th2 cytokine profile released by iNKT cells. Depending on the length and time of *in vivo *exposure to *α*-GalCer, this glycolipid strongly activates iNKT cells to secrete variable amounts of Th1 and/or Th2 cytokines. Studies have provided evidence for the ability of *α*-GalCer, when administered in repeated doses or coadministered with antigen, to polarize the iNKT cell cytokine response to a more Th2-like phenotype [40, 41], whereas others support Th1 polarization by *α*-GalCer [21, 42, 43]. Although the mechanism underlying these differences is unclear, the Th2 polarization may be due to a reported induction of iNKT cell anergy following injection of soluble *α*-GalCer [44, 45], characterized by a strong blunting in IFN-*γ* production and an inability of iNKT cells to respond to DC stimulation to produce IFN-*γ*.Further, bias towards a Th1 cytokine environment appears to be mediated by DC maturation and its presentation of *α*-GalCer [21, 42], whereas anergy may be induced by non-DC APCs [44, 45]. When used in a mouse model of the systemic Shwartzman reaction, treatment with *α*-GalCer prophylactically or shortly after LPS challenge protected mice against the systemic Shwartzman reaction [46, 47]. This protection was correlated with significantly lower serum levels of Th1 cytokines, including IFN-*γ* and TNF-*α*, as well as an increase in Th2 cytokines such as IL-10 [46, 47]. Protected animals also exhibited a higher frequency of iNKT cells positive for intracellular IL-10 and a lower frequency of iNKT cells positive for intracellular IFN-*γ* [46, 47]. These results support a potential therapeutic application for *α*-GalCer in modulating and shifting the iNKT cell response to a more anti-inflammatory phenotype. 

In less severe cases of endotoxemia and other microbial infections, the IFN-*γ* produced by iNKT cells has been shown to facilitate pathogen clearance [48]. In an endotoxemia model induced by a single intravenous injection of LPS, iNKT cells produced mostly IFN-*γ* and undetectable levels of IL-4 after LPS exposure [23]. In that study, iNKT cells appeared to contribute to endotoxemic mortality, because *J* *α*18^−/−^ mice deficient in iNKT cells showed greater survival in association with a strong reduction in IFN-*γ* levels [23]. In the same study, TNF decreased even more dramatically early after LPS challenge, which was attributed to insufficient induction by iNKT cell-derived IFN-*γ* [23]. Finally, NK cell activation and production of IFN-*γ* were also decreased in *J* *α*18^−/−^ mice, suggesting a role for iNKT cells in amplifying the immune response by rapidly activating other immune cell types [23]. All of these findings indicate that iNKT cells and their early synthesis of IFN-*γ* are critical to the complete activation of the pro-inflammatory cascade, which is important for the proper clearance of infection but may have deleterious consequences when overly activated.

## 5. Polymicrobial Sepsis

Although LPS is a fundamental factor in many cases of sepsis, criticism has arisen over the relevancy of experimental endotoxemia to clinical sepsis and the accuracy of translating experimental results to the treatment of septic patients [24]. Cecal ligation and puncture (CLP) is a well-established animal model of sepsis that closely replicates the features and clinical course of sepsis including polymicrobial peritonitis and the development of a hyperinflammatory state that may lead to a hyporesponsive phase [25]. The CLP procedure consists of ligation of a percentage of the cecum followed by perforation of the ligated portion, providing a constant source of fecal bacteria draining into the peritoneal cavity. Because CLP meets many of the criteria deemed necessary for an appropriate sepsis model, it is one of the most widely used tools to study the mechanisms and pathophysiology of sepsis and to test the efficacy and safety of pharmaceutical agents before clinical application.

Administration of anti-CD1d antibody has been demonstrated to ameliorate systemic lupus erythematosus, airway hyperreactivity in allergic asthma, and to exhibit potential antitumor activity [49–51]. Pretreatment with monoclonal antibody (mAb) blocking CD1d was shown to reduce CLP-induced mortality compared to IgG-treated controls and to suppress plasma and splenic levels of the Th2 cytokine IL-10 [52]. Other studies had postulated a role for NKT cells in the hyperinflammatory phase of sepsis, but this study suggested their contribution to the later immunosuppressed state [52]. However, there have been new studies demonstrating the agonistic ability of CD1d mAbs to enhance Th1 responses [50, 53], although these studies looked at conditions in which there is deficient protective immunity by iNKT cells and examined the ability of these antibodies to bypass iNKT activation and directly stimulate CD1d^+^ APCs. Another study showed that *β*2m^−/−^ mice had prolonged survival relative to wild-type mice after CLP but did not improve endpoint survival. Additional depletion of NK cells by anti-asialoGM1 antibody conferred complete resistance to septic mortality and was associated with diminished pro-inflammatory cytokine synthesis and secretion [26]. Similarly, *J* *α*18^−/−^ mice were used to show that iNKT cell deficiency significantly decreased septic mortality and ameliorated the systemic pro-inflammatory response [22]. Despite contradictory findings on the relative contribution of iNKT cells to a Th1 or Th2 response, these results consistently implicate a detrimental effect of NKT cell activation in polymicrobial sepsis.

## 6. Mechanisms of iNKT Cell Activation

The mechanism(s) by which iNKT cells are activated by microbial infection remains to be further elucidated. A “direct” pathway has been reported in which the TCR of iNKT cells recognizes the GSL cell-wall components of microbial pathogens, including *Sphingomonas* bacteria [54–56]. This early activation of iNKT cells appears to be important for bacterial clearance, because CD1d^−/−^ and *J* *α*18^−/−^  mice were impaired in their ability to clear *Sphingomonas *[55]. However, this proposed mechanism does not apply to all models of infection in which iNKT cells have been implicated. Thus, alternative pathways for activation by pathogens that do not possess an antigen recognized by the TCR must exist. 

Recent studies have reported an “indirect” pathway with live *Salmonella typhimurium* and *Salmonella* LPS infection [55, 57, 58]. This consists of a combination of two signals that culminates in potent IFN-*γ* secretion by iNKT cells. The first signal is a weak TCR-mediated response that is generated from the recognition of CD1d-presented endogenous GSL antigens. The requirement for self-antigen recognition was concluded from the observation that CD1d blocking mAb inhibited iNKT cell activation when cocultured with dendritic cells in the presence of *S. typhimurium *[57]. These weak responses are amplified by APC-derived IL-12, which stimulates the IL-12 receptor expressed by iNKT cells after TLR is activated by microbial products [57, 58]. IL-12 alone cannot activate iNKT cells when dendritic cells are absent, which provides further evidence that recognition of self-ligand is an essential part of this indirect pathway [57, 58].

Yet a third mechanism by which iNKT cells can be activated in response to bacteria is dependent only on the APC-derived cytokines IL-12 and IL-18. In iNKT cells from IL-12^−/−^  mice, production of IFN-*γ* was impaired in response to LPS, although not completely abrogated [23]. This impairment was even more significant with iNKT cells from IL-18^−/−^ mice, suggesting that the iNKT cell IFN-*γ* response requires early APC activation and their production of IL-12 and IL-18 [23]. CD1d-presented antigen was also shown to be unnecessary for iNKT cell activation because adding anti-CD1d Ab to cocultures of iNKT cells with dendritic cells and LPS had little effect on IFN-*γ* production, and dendritic cells from CD1d^−/−^ mice were just as effective in stimulating IFN-*γ* secretion as those from wild-type animals [23]. Furthermore, the addition of either recombinant IL-12 or IL-18 to iNKT cells alone was sufficient to induce a measurable amount of IFN-*γ* production, whereas stimulation with IL-12 and IL-18 combined, even at lower doses, had a synergistic effect and resulted in considerably enhanced IFN-*γ* response compared to incubation with cytokines individually [23]. Because there were comparable levels of IL-12 and IL-18 following LPS injection in *J* *α*18^−/−^  and wild-type mice, while there was a strong reduction in IFN-*γ* in *J* *α*18^−/−^ mice, it appeared that iNKT cells were acting downstream of APC-derived IL-12 and IL-18 and were being activated by these cytokines to produce IFN-*γ* [23].

All these pathways could potentially be at work in the pathophysiology of polymicrobial sepsis due to the systemic release of many microbial stimuli. The mechanism may be dependent on the nature and strength of the stimulus and the type of APC that is activated. In addition, these indirect pathways are postulated to be part of a positive feedback loop whereby the IFN-*γ* ultimately produced by iNKT cells feeds back to further activate APCs [23]. Nonetheless, these pathways all represent ways in which iNKT cells can quickly amplify the innate immune response to infection and contribute to the rapid development of the hyperinflammatory response in sepsis.

## 7. NKT versus NK Cells as Major Mediators of Septic Inflammation and Mortality

Some groups have reported that NK cells are more prominent mediators of septic inflammation and mortality than NKT cells given that NK cells are also known to be potent producers of IFN-*γ*. Mice treated with anti-asialoGM1 (NK cell deficient) or anti-NK1.1 (NK and NKT cell deficient) were both protected against CLP-induced mortality compared to IgG-treated controls, whereas CD1 knockout mice (NKT cell deficient) had no significant difference in survival compared to wild-type controls [59]. This survival benefit was associated with significantly lower serum concentrations of IL-6 and MIP-2 [59]. NK cell depletion also decreased the activation of peritoneal macrophages and other myeloid lineage cells [59]. Furthermore, NK cell numbers in the peritoneal cavity increased significantly in CLP versus sham mice, whereas cell numbers in the spleen and blood decreased [59]. Similarly, *β*_2_-microglobulin-deficient mice that lack iNKT cells, but not NK cells, as well as *J* *α*18^−/−^  mice were found to be more susceptible to LPS-induced shock than wild-type controls [60]. This led to the conclusion that NK cells rather than NKT cells contribute to septic inflammation, in part by migrating to the peritoneal cavity, where they upregulate the proinflammatory activities of certain myeloid cell populations [59]. 

Although NK cells are possibly the more immediate effector cells in inducing septic shock, NKT cells have been shown to play a key role in transactivating NK cells at a very high speed upon stimulation. The glycolipid *α*-GalCer was shown to rapidly activate NK cells to produce IFN-*γ* and upregulate their expression of the early activation marker CD69 within hours of exposure, but this effect was abrogated in CD1-deficient mice lacking NKT cells [61]. The great rate and magnitude at which NK cells are transactivated by activated NKT cells led to the postulate that NK cells are responding to the cytokines that are promptly secreted by stimulated NKT cells because pretreatment with anti-IFN-*γ* Ab partially reduced intracellular levels of IFN-*γ* and CD69 expression in NK cells [61]. This provides yet more evidence for the pivotal role of NKT cells as a bridge between innate and adaptive immunity in initiating an immune cascade that involves both compartments of the immune system.

## 8. Lipemia of Sepsis

Early evidence of lipoproteins functioning in innate immunity came from the observation that animals or humans challenged with infectious agents or LPS exhibit significant changes in the distribution of their circulating lipoproteins. This “lipemia of sepsis” was initially described in the late 1950s when patients with cholera were noted to have grossly lipemic blood and high serum levels of triglyceride (TG) [62]. The same phenomenon was later observed in patients experiencing polymicrobial infection [63]. Experimental studies have shown that the lipemia of sepsis is primarily due to the accumulation of very low density lipoproteins (VLDLs) [64]. Although this hyperlipoproteinemia could simply represent the mobilization of fat stores to fuel the increased metabolic demands of the host during infections, the ability of lipoproteins to directly interact with LPS and other bacterial, viral, and fungal toxins certainly indicates a function of these particles beyond lipid transport.

Despite a previous focus by others on the interactions between high density lipoproteins (HDLs) and LPS [65–67], our laboratory demonstrated that all classes of lipoproteins can bind and neutralize LPS both *in vitro *[68, 69], and *in vivo* [70], and that infusion with TG-rich lipid emulsion protected mice against LPS-induced death. Over the past decade, additional evidence has surfaced supporting a protective role for lipoproteins against LPS-mediated toxicity. In the various studies, increased circulating lipoprotein concentrations were induced via either (1) the infusion of exogenous lipoproteins [71–73], synthetic emulsion [70, 72], or recombinant lipoproteins [74–79], (2) genetic manipulation of the catabolic machinery of lipoproteins [80], or (3) diet [81]. Despite the differing experimental models, all studies consistently demonstrated a protective role for increased systemic concentrations of all classes of lipoproteins against LPS compared to normolipidemic controls. In addition, hypolipidemic animals exhibited an increased susceptibility to LPS toxicity that is reversed when plasma lipid levels are returned to normal range [71].

## 9. ApoE Is Protective in Endotoxemia

While the capacity of lipoproteins to bind LPS is accurately predicted by the phospholipid content of the lipid particles [75, 78], additional protein constituents also play important roles in the process, including apoE. ApoE, a component of triglyceride-rich lipoproteins and a ligand of the low density lipoprotein receptor (LDLR), has a well-established role in lipid metabolism and cardiovascular disease. Beyond this, this 299-amino acid, 34.2-kD glycoprotein synthesized mainly by the liver, but also in many other organs and tissues including macrophages, as well as the brain and kidney, is being increasingly recognized as a pleiotropic molecule that participates in several human diseases, including Alzheimer's, dementia, diabetes, breast cancer, multiple sclerosis, stroke, and kidney disease [82–84]. More recently, it has emerged as an important regulator of the host immune response to infection. Consistent with its ability to facilitate the clearance of TG-rich lipoproteins by the liver, apoE has been shown to both redirect LPS from Kupffer cells to hepatocytes, [79] and protect against endotoxemia in rats [85]. ApoE knockout mice are also more susceptible to endotoxemia and gram-negative infections despite their elevated plasma lipid concentrations [86]. In addition, intravenous apoE protected against endotoxemia *in vivo*, further suggesting anti-infective properties [87]. Taken together, these observations identify apoE as an essential component of lipoprotein-mediated protection against LPS.

## 10. ApoE Regulates the Host Septic Immune Response

**Figure 1:**
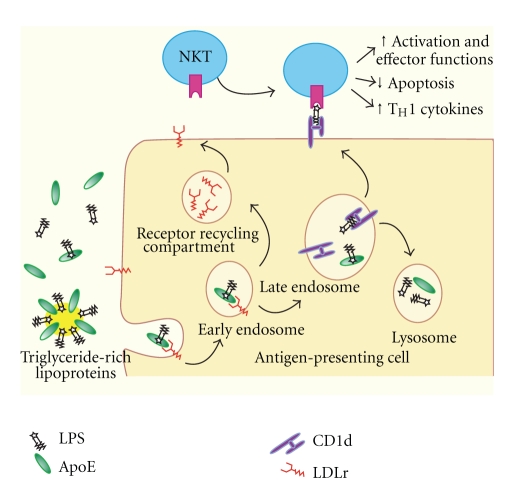
Proposed model of apoE-mediated lipid antigen presentation and NKT cell activation [89]. Pathogenic microbial lipids (i.e., lipopolysaccharide, LPS) are delivered to APCs as part of a triglyceride-rich lipoprotein or bound to apoE. The lipid complex binds to the LDLR, is internalized, and while trafficked through the endosomal compartments, is processed and loaded onto CD1d for recognition by NKT cells.

Contrary to published evidence documenting the protective effects of apoE, our laboratory found that injections of apoE increased CLP-induced septic morbidity and mortality in a dose-dependent manner [88]. This discrepancy between the literature and our findings could be attributed to differences in the experimental models, since the predominant model is a single injection of one type of endotoxin and a single dose of apoE, whereas our model uses continuous release of various types of fecal bacteria and four consecutive doses of apoE. These seemingly discordant findings led us to hypothesize that apoE regulates the host response to severe infection via its effects on NKT cell activation and the resultant cytokine secretion. In support of our findings, recent studies have shown that, in human serum, apoE is the major factor that mediates the presentation of lipid antigens to CD1-restricted NKT cells and that apoE specifically enhances T cell responses to a broad range of microbial lipids through an LDLR-dependent mechanism [89]. Therefore, we proposed that apoE is fueling iNKT cell activation and promoting septic mortality by acting as a molecular chaperone for bacterial antigens, targeting them for uptake by APCs and the subsequent processing and presentation of pathogenic lipid fragments to NKT cells in conjunction with CD1d (Figure 1). 

Indeed, we found that the apoE-mediated increase in septic mortality was also associated with increased splenic and hepatic iNKT cell frequencies, proliferation and cell numbers, and an exacerbated degree of liver injury [88]. Consistent with the pro-inflammatory contribution that apoE may be making in sepsis, hypomorphic apoE mice (Apoe^*h*/*h*^) expressing 2–5% of wild-type serum levels of apoE [90] were found to be less susceptible to septic mortality than their induced (wt apoE levels) counterparts [unpublished data]. Higher apoE levels were also associated with increased Th1 cytokine secretion, as the serum cytokine profile showed a trend towards greater Th1 cytokine concentrations (TNF, IL1-*β*, and IFN-*γ*) in septic, induced mice compared to septic, noninduced (low apoE) mice [unpublished data]. Furthermore, it appears that the physiological concentration of apoE may be regulating a delicate balance point between sufficient systemic inflammation to clear the invading pathogen, yet insufficient to harm the host in the process of eradicating the infection.

## 11. ApoE Polymorphisms Affect Septic Outcome

The APOE gene codes for three main isoforms, apoE2, E3, and E4, only differ from each other by a single amino acid substitution, but possess differential binding affinities for the LDLR (E4 > E3 ⋙ E2) [91]. Recent evidence has implicated apoE polymorphisms in modulating the septic inflammatory response and thus, influencing septic morbidity and outcome. Specifically, in patients, the apoE3 allele has been associated with a lower incidence of sepsis after elective surgery than the apoE4 allele [92]. In addition, after LPS exposure, mice expressing human apoE4 produced more IL-6 and TNF-*α* than mice expressing human apoE3 [93]. Furthermore, apoE4 mice were shown to be more susceptible to CLP-induced septic mortality than apoE3 mice, and this was associated with enhanced pro-inflammatory cytokine production [94]. That same study also showed that apoE-mimetic peptides that bind and block the apoE-binding region of LDLR reduced septic mortality and pro-inflammatory cytokine levels [94]. Similarly, our laboratory has demonstrated that treatment with heparin, shown to bind and inhibit the LDLR-binding region of apoE [95], promotes survival after CLP-induced sepsis [unpublished data]. Taken together, these findings provide further support for our hypothesis that apoE promotes septic mortality and a Th1 cytokine response by NKT cells via a mechanism mediated by LDLR. In particular, the apoE3 allele and mimetic peptides are linked to improved septic outcome because in both cases, there is decreased binding of apoE to LDLR and possibly inhibited delivery of bacterial antigens to APCs for presentation to iNKT cells and subsequent activation.

## 12. ApoE May Enhance Presentation of Endogenous Lipid Antigens

Despite evidence that iNKT cells are activated in response to LPS *in vivo *[23], apoE failed to enhance iNKT cell activation when added to cocultures containing purified LPS or heat-killed bacteria containing LPS [96]. Instead, apoE significantly increased iNKT cell activation in response to the endogenous ligand analog iGb3 [96]. These *in vitro* findings suggest that apoE-mediated antigen presentation is more prominent with endogenous ligands than with LPS, although the classic TLR4 pathway of direct LPS recognition may play a more substantial role in inducing an inflammatory response *in vivo*. Although the primary insult in CLP appears to be the systemic release of LPS, or other pathogen-associated molecular patterns (PAMPs), and induction of fecal peritonitis, the traumatic ligation and puncture of the cecum could result in the release of endogenous ligands or danger-associated molecular patterns (DAMPs) that can contribute significantly to inflammation as well. The contribution of DAMPs to dendritic cell and iNKT cell activation is not unprecedented, having been studied in attempts to develop immunotherapies against malignancies [97]. This evidence potentially expands apoE's role in promoting septic mortality to include mediating the presentation of not only PAMPs, but DAMPs as well, in a CLP model of sepsis. In light of failed clinical trials of anticytokine and anti-LPS treatments, [18, 98] apoE antagonism may hold more promise in conferring protection against bacterial sepsis since it can potentially act on two separate classes of molecules that initiate and perpetuate the host septic immune response.

## 13. Conclusions

Despite different experimental models, the studies reviewed here all constitute growing evidence for the large contribution of NKT cells to the dysregulated and overwhelming pro-inflammatory response in polymicrobial sepsis and endotoxic shock. Although many strong correlations have been made between septic mortality and NKT cell activation and cytokine production, researchers are still far from delineating and demonstrating the exact mechanism by which NKT cells participate in the septic immune response, or how their activity is regulated in general. In order to accomplish this, knowledge of the endogenous ligand(s) of NKT cells is needed. In spite of progress made by the identification of iGb3, the self-ligand(s) is still unknown, and investigators anxiously await the discovery of this missing piece of the puzzle.

 Considering the ability of NKT cells to bridge innate and adaptive immunity and their extensive immunoregulatory roles, manipulation of these cells provides a promising therapeutic strategy for sepsis and inflammation. However, because NKT cells have numerous effector functions and closely communicate with other cell types, it will be a challenge to direct the effect of these interventions to a single mechanism of action. In promoting the onset of sepsis, there is contradictory evidence of whether NKT cells are deleterious due to their pro-inflammatory or anti-inflammatory contribution. To further tailor therapies that modulate NKT cell activity, more work is needed to determine what specific factors trigger NKT cells to assume a Th1 or Th2-like phenotype during sepsis. Nonetheless, the work done in animal models so far provides insight on the contribution of NKT cells to inflammation and injury, and an important foundation upon which to build more targeted therapies.

## Figures and Tables

**Table 1:** Summary of proposed role of NKT cells in experimental models of sepsis.

Experimental model	Role of NKT Cells	Reference
Generalized Schwartzman reaction	Deleterious: anti-NK1.1 Ab-treated, *β*2m^−/−^ and *J* *α*281^−/−^ mice were protected	[19, 20]

Endotoxemia (single i.v. LPS injection)	Deleterious: *J* *α*18^−/−^ mice were protected	[21]
	No role: *β*2m^−/−^ and *J* *α*18^−/−^ mice were more susceptible to LPS-induced shock	[22]

Polymicrobial sepsis (cecal ligation and puncture)	Deleterious:	
	(a) pretreatment with anti-CD1d blocking Ab decreased septic mortality	[23]
	(b) *β*2m^−/−^ mice treated with anti-asialo GM1 Ab and *J* *α*18^−/−^ mice had improved survival	[24, 25]
	No role: CD1-KO mice exhibited no significant difference in survival; mice depleted of NK cells were protected	[26]
